# Calcium handling maturation and adaptation to increased substrate stiffness in human iPSC-derived cardiomyocytes: The impact of full-length dystrophin deficiency

**DOI:** 10.3389/fphys.2022.1030920

**Published:** 2022-11-07

**Authors:** Josè Manuel Pioner, Lorenzo Santini, Chiara Palandri, Marianna Langione, Bruno Grandinetti, Silvia Querceto, Daniele Martella, Costanza Mazzantini, Beatrice Scellini, Lucrezia Giammarino, Flavia Lupi, Francesco Mazzarotto, Aoife Gowran, Davide Rovina, Rosaria Santoro, Giulio Pompilio, Chiara Tesi, Camilla Parmeggiani, Michael Regnier, Elisabetta Cerbai, David L. Mack, Corrado Poggesi, Cecilia Ferrantini, Raffaele Coppini

**Affiliations:** ^1^ Department of Biology, University of Florence, Florence, Italy; ^2^ Department of Neurofarba, University of Florence, Florence, Italy; ^3^ Department of Experimental and Clinical Medicine, University of Florence, Florence, Italy; ^4^ European Laboratory for Non-Linear Spectroscopy (LENS), University of Florence, Florence, Italy; ^5^ Istituto Nazionale di Ricerca Metrologica (INRiM), Turin, Italy; ^6^ Department of Molecular and Translational Medicine, University of Brescia, Brescia, Italy; ^7^ National Heart and Lung Institute, Imperial College London, London, United Kingdom; ^8^ Unit of Vascular Biology and Regenerative Medicine, Centro Cardiologico Monzino IRCCS, Milan, Italy; ^9^ Department of Biomedical, Surgical and Dental Sciences, University of Milan, Milan, Italy; ^10^ Department of Chemistry “Ugo Schiff”, University of Florence, Florence, Italy; ^11^ Department of Bioengineering, University of Washington, Seattle, WA, United States

**Keywords:** human iPSC derived cardiomyocytes, dystrophin (DMD), substrate stiffness, calcium handing, duchenne muscular dystrophy (DMD)

## Abstract

Cardiomyocytes differentiated from human induced Pluripotent Stem Cells (hiPSC- CMs) are a unique source for modelling inherited cardiomyopathies. In particular, the possibility of observing maturation processes in a simple culture dish opens novel perspectives in the study of early-disease defects caused by genetic mutations before the onset of clinical manifestations. For instance, calcium handling abnormalities are considered as a leading cause of cardiomyocyte dysfunction in several genetic-based dilated cardiomyopathies, including rare types such as Duchenne Muscular Dystrophy (DMD)-associated cardiomyopathy. To better define the maturation of calcium handling we simultaneously measured action potential and calcium transients (Ca-Ts) using fluorescent indicators at specific time points. We combined micropatterned substrates with long-term cultures to improve maturation of hiPSC-CMs (60, 75 or 90 days post-differentiation). Control-(hiPSC)-CMs displayed increased maturation over time (90 vs 60 days), with longer action potential duration (APD), increased Ca-T amplitude, faster Ca-T rise (time to peak) and Ca-T decay (RT50). The progressively increased contribution of the SR to Ca release (estimated by post-rest potentiation or Caffeine-induced Ca-Ts) appeared as the main determinant of the progressive rise of Ca-T amplitude during maturation. As an example of severe cardiomyopathy with early onset, we compared hiPSC-CMs generated from a DMD patient (DMD-ΔExon50) and a CRISPR-Cas9 genome edited cell line isogenic to the healthy control with deletion of a G base at position 263 of the DMD gene (c.263delG-CMs). In DMD-hiPSC-CMs, changes of Ca-Ts during maturation were less pronounced: indeed, DMD cells at 90 days showed reduced Ca-T amplitude and faster Ca-T rise and RT50, as compared with control hiPSC-CMs. Caffeine-Ca-T was reduced in amplitude and had a slower time course, suggesting lower SR calcium content and NCX function in DMD vs control cells. Nonetheless, the inotropic and lusitropic responses to forskolin were preserved. CRISPR-induced c.263delG-CM line recapitulated the same developmental calcium handling alterations observed in DMD-CMs. We then tested the effects of micropatterned substrates with higher stiffness. In control hiPSC-CMs, higher stiffness leads to higher amplitude of Ca-T with faster decay kinetics. In hiPSC-CMs lacking full-length dystrophin, however, stiffer substrates did not modify Ca-Ts but only led to higher SR Ca content. These findings highlighted the inability of dystrophin-deficient cardiomyocytes to adjust their calcium homeostasis in response to increases of extracellular matrix stiffness, which suggests a mechanism occurring during the physiological and pathological development (i.e. fibrosis).

## Introduction

Recent evidence suggested that abnormalities of cardiomyocyte maturation and cardiac muscle development contribute to functional and structural cardiac anomalies in genetically-determined cardiomyopathies (Girolami et al., 2022). Cardiomyopathy-related mutations may alter the function of the developing cardiac cells determining pathological changes that may persist in the adult heart (Olivotto et al., 2009a). The multisystemic and heterogeneous presentation of many types of inherited cardiomyopathies is a challenge for clinicians, and delay in diagnosis is a significant concern (Limongelli et al., 2022). As an example, a number of cardiac abnormalities, including mitral valve leaflet elongation and coronary vessel wall thickening, may be the consequence of developmental abnormalities (Olivotto et al., 2009b; Monteiro da Rocha et al., 2016; Krane et al., 2021). However, studying the abnormalities of cardiac development at cell level in transgenic rodent cardiomyopathy models has many limitations and concerns, due to the large differences in cardiac embryonic maturation between mice and humans. Patient-specific induced pluripotent stem-cells differentiated into cardiomyocytes (hiPSC-CMs) are at the Frontier for *in vitro* modelling of genetic cardiac diseases and reproduce several disease-specific pathological changes observed in human hearts (Marchiano et al., 2019). Notably, differentiation from hiPSC and maturation of hiPSC-CMs summarize the changes occurring in the cardiac muscle during the embryonic and fetal period. However, most studies used early stage (30–45 days post-differentiation) hiPSC-CMs and the longer time-point of maturation were not monitored. We previously showed that control hiPSC-CMs grown on surfaces with microgrooved topography for an extended culture time (60-75-90 days) can regularly follow an external pacing frequency and more closely reflect the functional properties of native human cardiomyocytes from healthy hearts (Pioner et al., 2019a). The first issue that this work seeks to address are the possible effects of cardiomyopathy-associated mutations on the cardiac differentiation and maturation of hiPSC-CM lines. Particularly, calcium handling has a prominent role in cardiomyocyte development. Immature cardiomyocytes lack transverse (T)-tubules and have a poorly organized sarcoplasmic reticulum (SR). For this reason, in the early phases after differentiation, calcium diffusion in the intracellular compartments is slower compared to later-stages of cardiomyocyte development (Lundy et al., 2013). Moreover, calcium fluxes rise before the development of the myofibril contractile apparatus and alterations of calcium handling during the development may strongly influence the mechanical function of mature cardiomyocytes (Pioner et al., 2020).

A second issue that is often overlooked in current hiPSC-CM research are the effects of the different culture substrates on cardiomyocyte structure and function (Querceto et al., 2022). Increased substrate stiffness and anisotropic surfaces morphology have both shown to be valuable biomimetic tools for advancing the structural and functional maturation of immature cardiomyocytes in culture (Querceto et al., 2022). Specifically, cardiomyocytes increase their contraction force in response to increased stiffness *via* adaptation of calcium handling and myofibril structural organization (Rodriguez et al., 2011; Ribeiro et al., 2015; Ribeiro et al., 2020). Morphological anisotropy of culture surfaces leads to a more rapid elongation of cardiomyocytes, improves myofibril alignment and increases sarcomere length (Carson et al., 2016). hiPSC-CMs are immature and beat spontaneously for a long time in culture. In our previous work, we showed that micropatterned surfaces with alternated linear grooves and ridges strongly impacted on cardiomyocyte regulation of calcium homeostasis and cellular electrophysiology (Pioner et al., 2019a). However, effects of substrates stiffness on hiPSC-CM function are rarely assessed. Understanding and translating the impact of substrate features on hiPSC-CM maturation is indeed highly relevant for *in vitro* cardiomyopathy modelling to avoid under- or over-estimation of mutation-related effects in studies involving patient-specific hiPSC-CMs. Additionally, changes of extracellular stiffness may occur in cardiomyopathies (e.g. due to accumulation of myocardial fibrosis) and severely affect cardiomyocyte function.

To highlight the possible implications of developmental anomalies and substrate changes for cardiomyopathy studies with hiPSC-CMs, we compared cardiomyocytes from a Duchenne Muscular Dystrophy (DMD) patient carrying a deletion of exon 50 in the *DMD* gene (Guan et al., 2014) with control cell lines and with an isogenic control created by CRISPR-Cas9 targeting the *DMD* gene (c.263delG) (Pioner et al., 2019b). DMD-CMs were chosen over other cardiomyopathy models because the lack of full-length dystrophin in diseased cardiomyocytes may directly affect the interaction of cardiac cells with the extracellular environment (Pioner et al., 2020). Therefore, the final aim of the study is to assess: (i) the effects of the lack of dystrophin on the maturation of intracellular Ca handling during development and (ii) the effects of substrate stiffness on the function of hiPSC-CM with and without dystrophin, by growing cells on micropatterned surfaces with different mechanical properties.

## Results

### Analysis of Ca-transient amplitude during cardiomyocyte maturation

**FIGURE 1:**
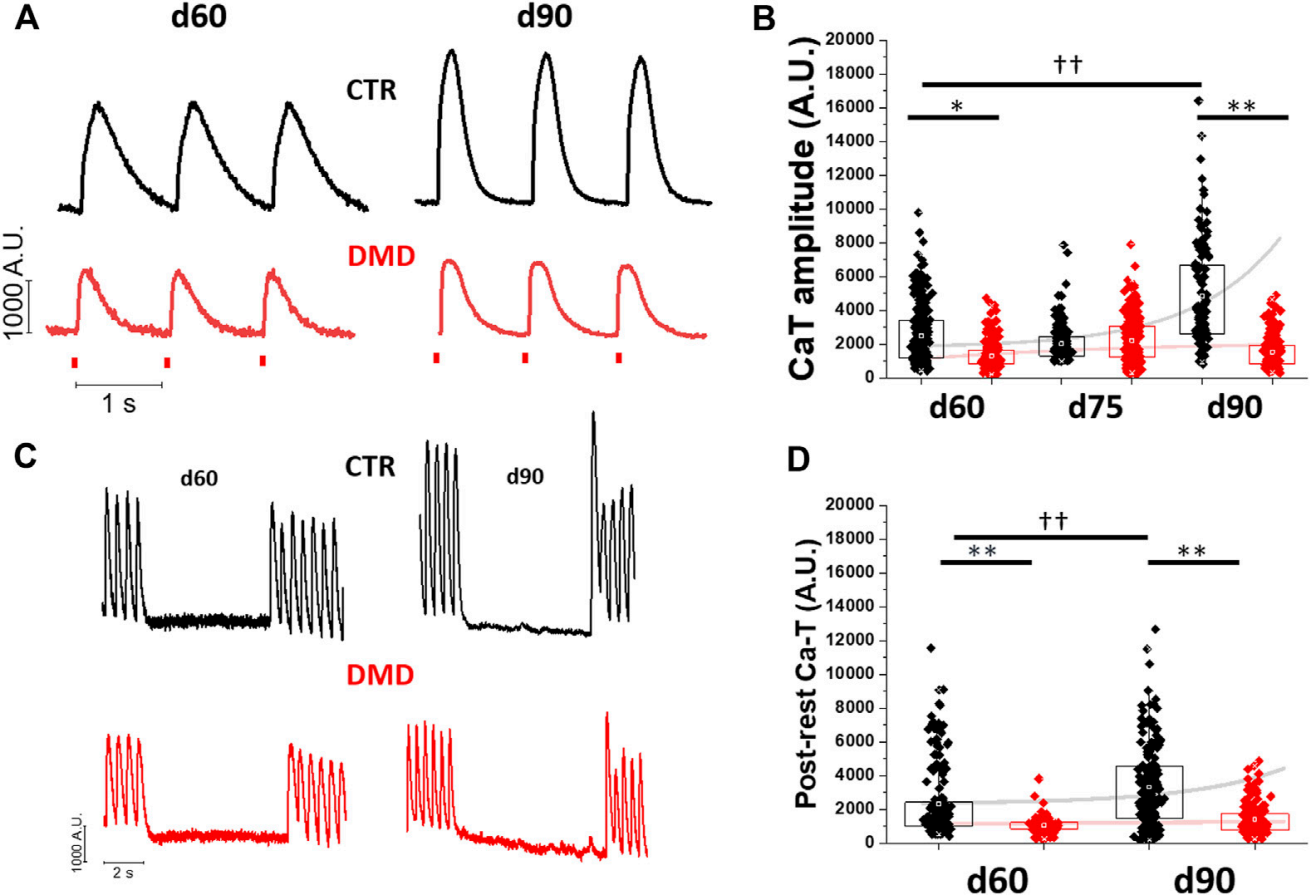
Changes of calcium transient amplitude during hiPSC-CM maturation. Calcium transients were estimated at day 60, 75 and 90 post differentiation at 37°C, 1.8 mM [Ca^2+^]. **(A)** Representative CaT profiles at day 60 and 90 and average CaT amplitude (Fluorescence Arbitrary Units, A.U.) of DMD-versus control-CMs at day 60,75 and 90. **(B)** Sarcoplasmic reticulum (SR) contribution in calcium handling maturation was tested by a post rest potentiation protocol at multiple maturation time-points. **(C)** The post-rest potentiation of CaT amplitude was estimated after a resting pause of 5 s, inserted in a regular train of stimulation at 2 Hz. **(D)** The potentiation is expressed as the % of increase of the first post-rest CaT with respect of CaT pacing train before the pause (%). Post rest potentiation of DMD-versus control-CMs is estimated at day 60 and day 90. Exponential curves with Stirling’s approximation were used to show the variation of maturation in both groups. Control d60 N = 3, n = 336; d75 N = 5, n = 251; d90 N = 3, n = 165; DMD d60 N = 3, n = 193, d75 N = 4, n = 292; d90 N = 4, n = 169. Supporting information given in Supplementary Table S2. One-way analysis of variance (ANOVA) with a Tukey post-hoc test with statistical significance set at ††*p* < 0.01 versus time point; *p < 0.05 and **p 0.01 versus control-CMs.

Single control- and DMD-(hiPSC)-CMs were dissociated from beating monolayers after differentiation (day 15) and plated onto custom-made photopolymerized polyethylene glycol-diacrylate (PEG-DA 100%) surfaces with a micro-structured topography. The geometry of this biomimetic substrate featured an array of parallel micro-grooves with 0.6 μm width, 1.5 μm depth and 1.4 μm distance between lines (Pioner et al., 2019a). To verify differences in the maturation of calcium handling, we compared control and DMD-CMs at different stages of cardiac differentiation (at day 60, 75 and day 90 from the beginning of cardiac differentiation, Figure 1A and Supplementary Table S1). In control iPSC-CMs, calcium transient (Ca-T) amplitude increased significantly during maturation from d60 to d90 (Figure 1B). In DMD-CMs vs controls, however, Ca-T amplitude was smaller at d60 and failed to increase at d90 (Figure 1B). We then used a post-rest potentiation protocol to assess calcium storage in the sarcoplasmic reticulum (SR) of iPSC-CMs, as previously described (Pioner et al., 2019b). Rest periods of 10 s were introduced during 2 Hz stimulation and the amplitude of the post-rest potentiated contraction was plotted against that of the last regular contraction during regular pacing (Figure 1C). In control cells, post-rest potentiation was larger at d90 vs d60, indicating an increase of SR Ca storage. In contrast, the potentiation of Ca-T in DMD-CMs was lower compared to control-CMs and did not significantly change from d60 to d90 (Figure 1D). Similarly, these results were recapitulated in age-matched CRISPR-Cas9 edited control cardiomyocytes lacking full-length dystrophin (c.263delG-CMs, Supplementary Figure S1).

### Changes of action potential and calcium transient kinetics during cardiomyocyte maturation

**FIGURE 2:**
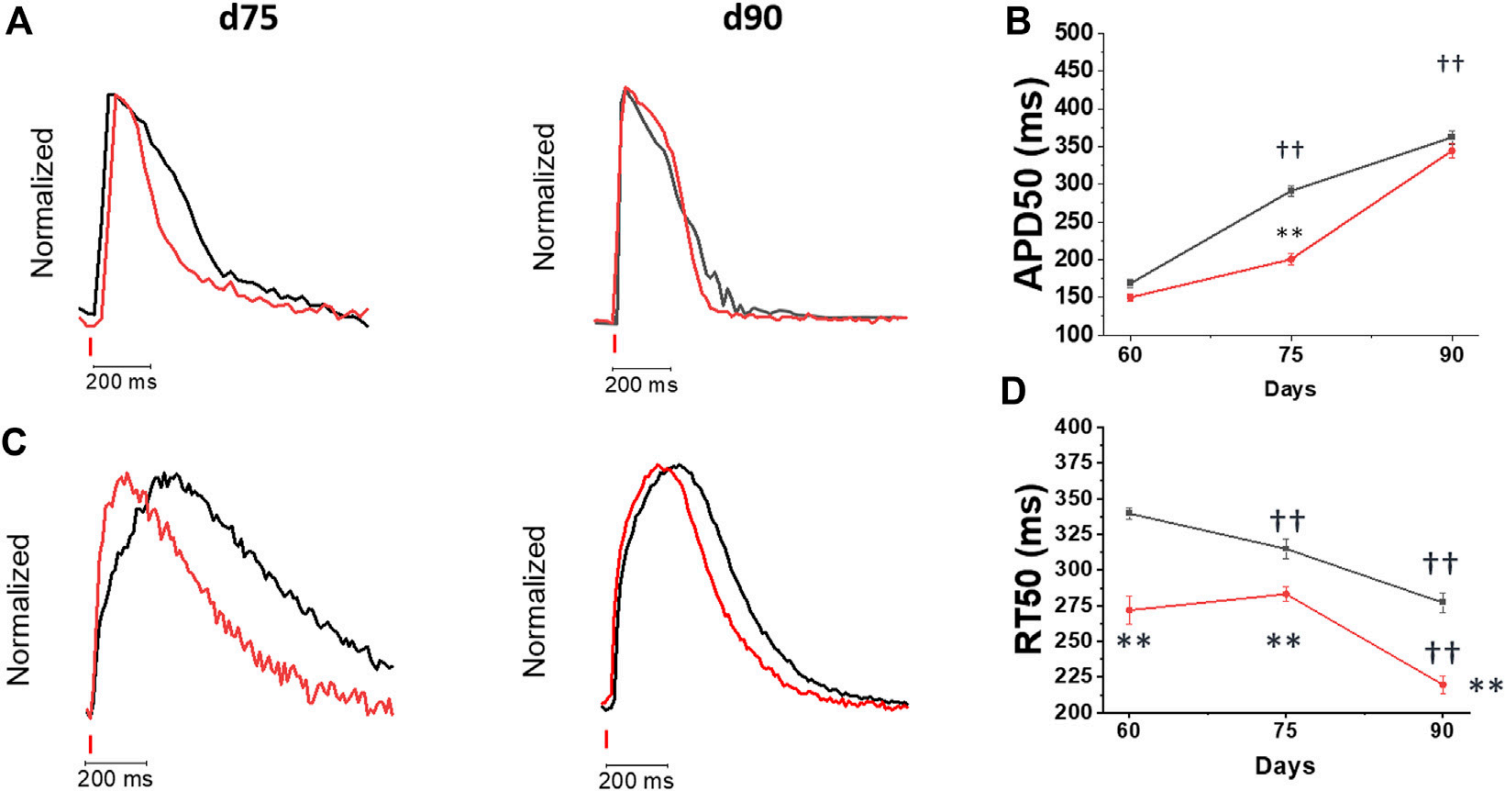
Changes of action potential and calcium transient kinetics during hiPSC-Cm maturation. Dual recording of action potential and calcium transients was performed at day 60, 75 and 90 post differentiation at 37°C at and external [Ca^2+^] = 1.8 mM on the softer substrate (PEG). **(A)** Superimposed action potential (AP) traces of day 75 (Control d75 N = 2; *n* = 186; DMD d75 N = 2; n = 91) vs day 90 (Control N = 2; *n* = 119; DMD: d90 N = 2; *n* = 44) recorded by FluoVolt **(B)** 50% of action potential durations (APD50, ms) at 1 Hz are reported as Mean ± SEM. **(C)** Superimposed traces of calcium transients recorded by Cal630 at day 60, 75 and 90 **(D)** average calcium transient (CaT) (difference of 50% of CaT decay and TTP, RT50, ms) are reported, during pacing at 1 Hz. Control d60 N = 3, *n* = 336; d75 N = 5, *n* = 251; d90 N = 3, *n* = 165; DMD d60 N = 3, n = 193, d75 N = 4, *n* = 292; d90 N = 4, *n* = 169. Supporting information given in Table S2. One-way analysis of variance (ANOVA) with a Tukey post-hoc test with statistical significance set at ††*p* < 0.01 versus time point; *p < 0.05 and **p 0.01 versus control-CMs.

In addition to amplitude, the kinetics of Ca-T under the influence of the kinetics of action potentials (APs) can greatly influence cardiomyocyte contractile properties. Therefore, we then analyzed the kinetics of both phenomena. APs were measured by acquiring the FluoVolt voltage-sensitive dye fluorescence together with Ca-T (Cal630 fluorescence), during regular pacing with field-stimulation at 1 Hz (Figure 2). Representative traces simultaneously recorded at day 75 and 90 post cardiac induction are reported in Figure 2A. In both control and DMD-CMs, AP duration (APD) increased during maturation (Figure 2B). At day 75, APD in DMD-CMs was shorter as compared with controls. At d90, however, no differences in APD50 were observed in DMD vs control cells (Figure 2B). Interestingly, in both control and DMD cells, APD did not show a significant rate adaptation at day 75 (APD50 at 1 and 2 Hz were comparable) but the shortening of APD with increased pacing rate (1 vs 2 Hz) was observed at d90 (Supplementary Figure S2). Ca-T kinetics was also measured at each time point of maturation (Figures 2C,D). In control hiPSC-CMs, the kinetics of Ca-Ts accelerated from d60 to d90. Similarly, in DMD-CMs, the kinetics of calcium rise (time to peak, TTP) and decay (time from peak to 50% of CaT decay, RT50) became more rapid after 90 days in culture (Supplementary Table S1; Figure 4D). However, the kinetics of Ca-Ts were consistently faster in DMD vs. controls myocytes (Figure 2D; Supplementary Table S2).

### Preserved response to forskolin in DMD-CMs

**FIGURE 3:**
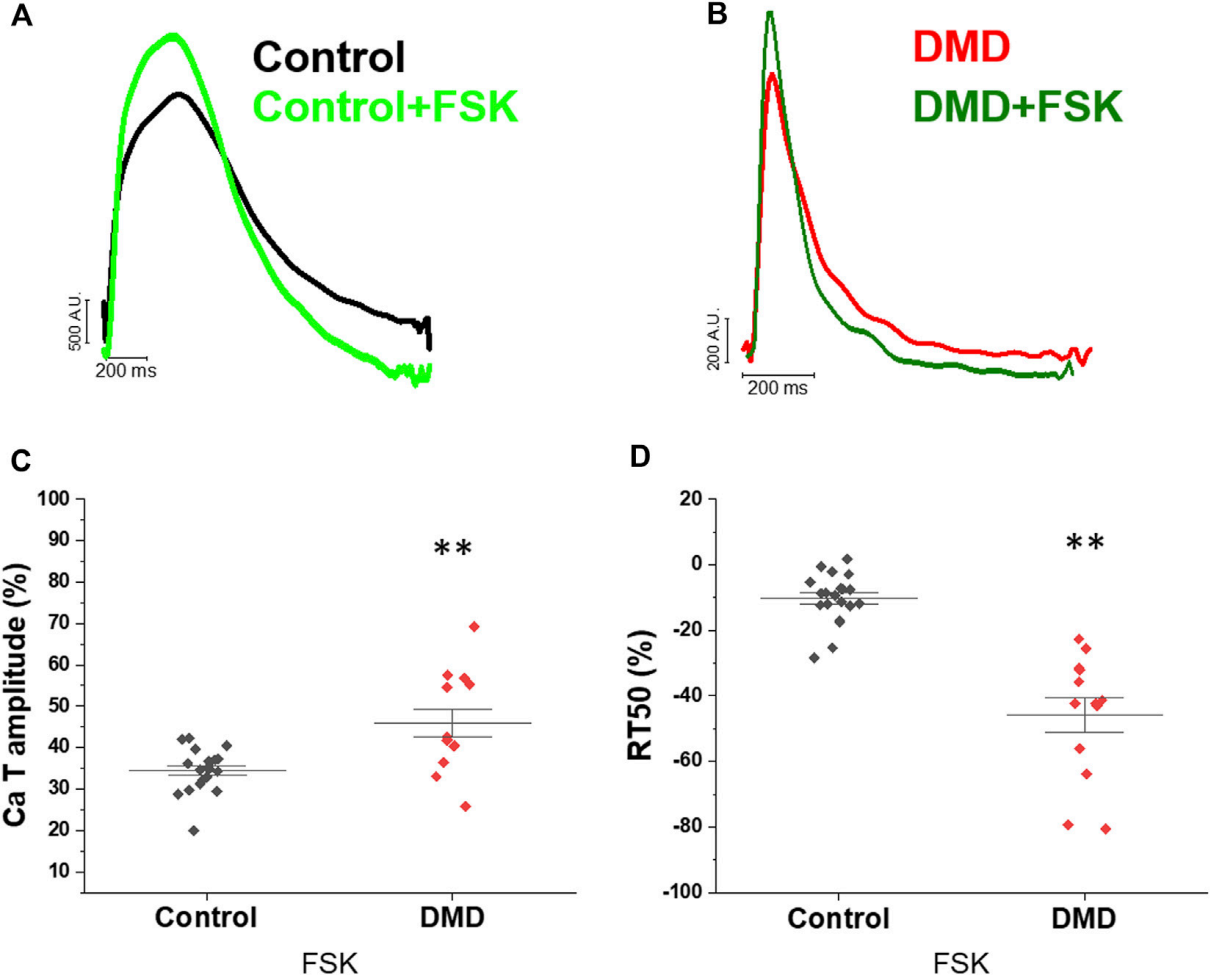
Effects of forskolin on calcium transients. Calcium transients of the before and after forskolin addition in day 60 control- and DMD-hiPSC-CMs. **(A,B)** Superimposed normalized traces of calcium transients recorded in the control hiPSC-CMs line, before and after treatment with forskolin (FSK, light green controls, dark green DMD), reported as the variation (%) compared with the basal condition. **(C)** Calcium transient amplitude is reported, during pacing at 1 Hz, as the % variation related to the basal condition compared to the treatment with FSK in DMD. **(D)** CaT decay (difference of 50% of CaT decay and TTP, RT50, ms) is reported, during pacing at 1 Hz, as the % variation related to the basal condition compared to the treatment with FSK in controls and DMD.

To investigate the response to the β-adrenergic pathway activation, hiPSC-CMs were acutely exposed to forskolin (FSK), a phosphodiesterase inhibitor, to increase cAMP levels and activate PKA. Figure 3A, B shows representative traces of Ca-Ts at baseline and with FSK, recorded in control and DMD-CMs respectively. In both DMD- and control-CMs, CaT amplitude increased by around 30% following FSK incubation compared to the basal condition (Figures 3C,D), suggesting preserved positive inotropic responses to catecholaminergic stimuli in DMD-CMs. In addition, the acceleration of RT50 with forskolin in both cell lines suggested a positive lusitropic response to beta-adrenergic stimulation (Figures 3E,F).

### CaMKII and RyR2 phosphorylation

**FIGURE 4:**
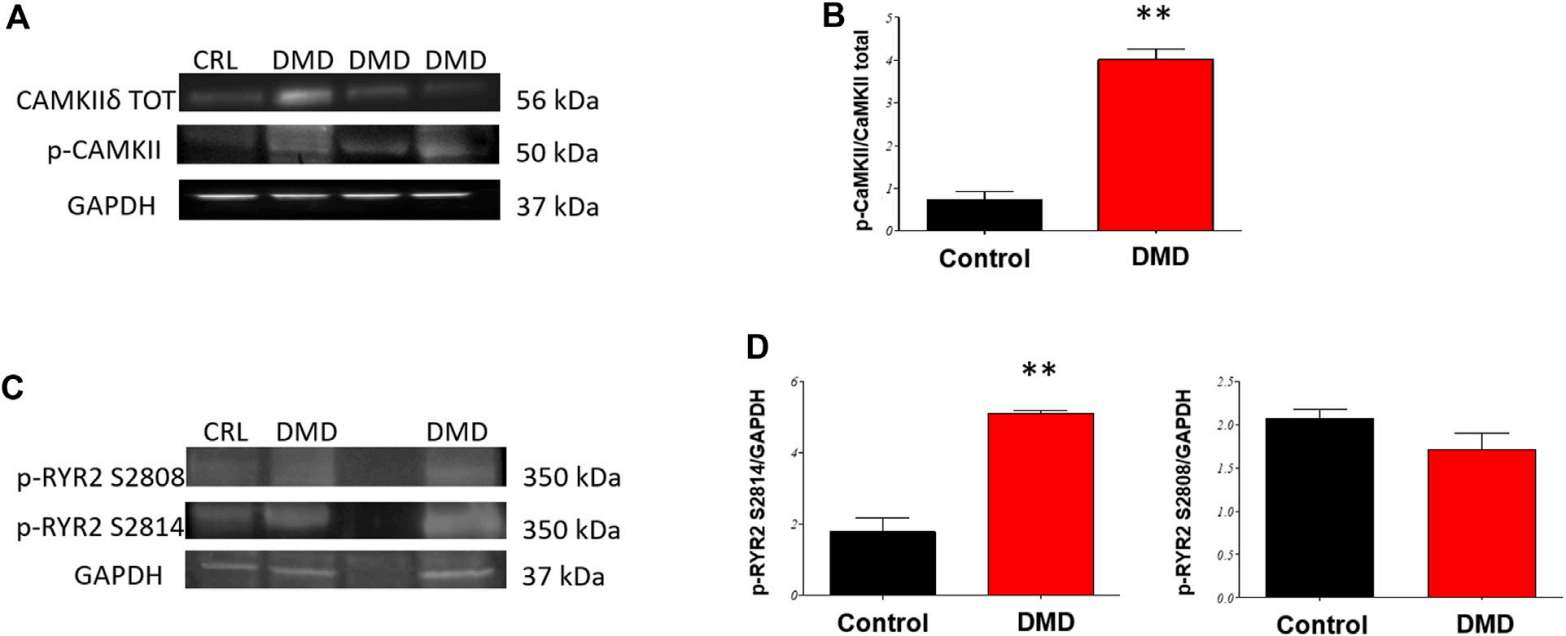
Phosphorylation of RyR2 and CaMKIIδ in hiPSC-CMs. Western blot (WBs) analysis of Ryanodine receptors (RyR2) phosphorylation status and verification of CaMKII activity in day 90 DMD-vs Control-hiPSC monolayers. **(A)** Representative WB of CaMKIIδ **(B)** and analysis normalized for total CaMKII. **(C)** Representative WB analysis of **(D)** RyR2 sites of phosphorylation (phospho-specific anti Ser 2814 target of CaMKII and Ser 2808 target of PKA) normalized for GAPDH, *p < 0.05 in ANOVA DMD vs Controls N = 3 (differentiation runs) and *n* = 2–5 (gel repetitions).

At later-stages (d90), DMD-CMs have a higher level of phosphorylated CaMKII compared to controls, indicating increased CaMKII activity (Figures 4A,B). We assessed the relative abundance of two phosphorylations (p-) sites of the ryanodine receptors (RyR2) using antibodies against p-RyR S2814 and p-RyR S2808, which are targets of CaMKII and PKA, respectively (Figures 4C,D). CaMKII-specific phosphorylation at site S2814 was increased in DMD-CMs compared to controls (*p* < 0.01). Contrarily, RyR2 phosphorylation at the PKA site (S2808) was not affected (Figures 4C,D). Increased phosphorylated RyR2 is associated with increased diastolic open probability, often associated with SR calcium leakage. This finding may partially account for reduced Ca-T amplitude of DMD-CMs.

### Stiffer substrates increase calcium transient amplitude in control hiPSC-CMs

**FIGURE 5:**
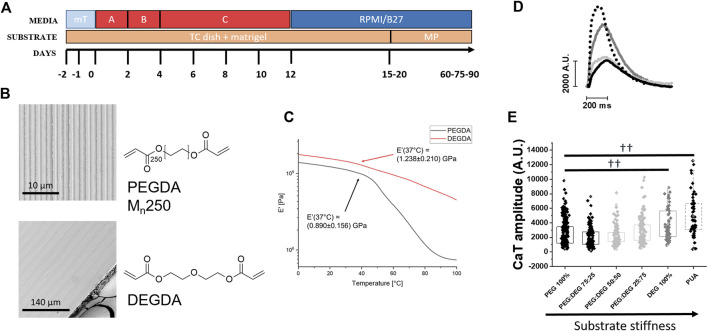
Ca-transients in DMD-hiPSC-CMs grown on PEG vs DEG patterned substrates. **(A)** Time course of media (mT mTeSR + suppl.; A + B + C cardiac differentiation kit) and substrates (TC tissue plate; MP micropatterned substrates). **(B)** S.E.M. image of Poly (ethylene glycol) diacrylate (PEG-DA)- and Di(ethylene glycol) diacrylate (DEG-DA)-based substrates with micropatterned grooves. Scale bars 10 μm. **(C)** Samples of PEGDA and DEGDA for dynamical mechanical analysis (DMA) with 1% wt. The samples were clamped in tension mode in a DMA800 analyzer (Perkin Elmer). Samples were analyzed in strain-control mode, imposing a 0.020 mm strain with 1 Hz frequency, in the -20°C-120°C temperature range. The mechanical characterization was repeated three times for PEGDA100 and DEGDA100 samples. The value of the storage modulus at E′ was obtained by calculating the integral mean of the E′-T curve between 36°C and 38°C. Values are expressed as mean ± SEM. **(D)** The impact of substrate stiffness in normal control-CMs was tested for CaT amplitude on microgrooved surfaces with increasing ratio of polyethyleneglycole (PEG) and dyethylenglycole (DEG) concentration and polyurethane-based nanopatterned surfaces (PUA) at 37 °C at and external [Ca^2+^] = 1.8 mM. Representative CaT profiles at day 60 and of CaT amplitude (Fluorescence Arbitrary Units, A.U.) of control-CMs under PEG (N = 3; *n* = 336), PEG:DEG 75:25 (N = 2; *n* = 150), 50:50 (N = 2; *n* = 147), 25:75 (N = 2; *n* = 150), DEG (N = 2; *n* = 50) and PUA (N = 2; n = 59). **(E)** Data were represented as a box (median [interquartile range]) and whisker plots. †*p* < 0.05, ††*p* < 0.01 versus control condition (PEG).

**FIGURE 6:**
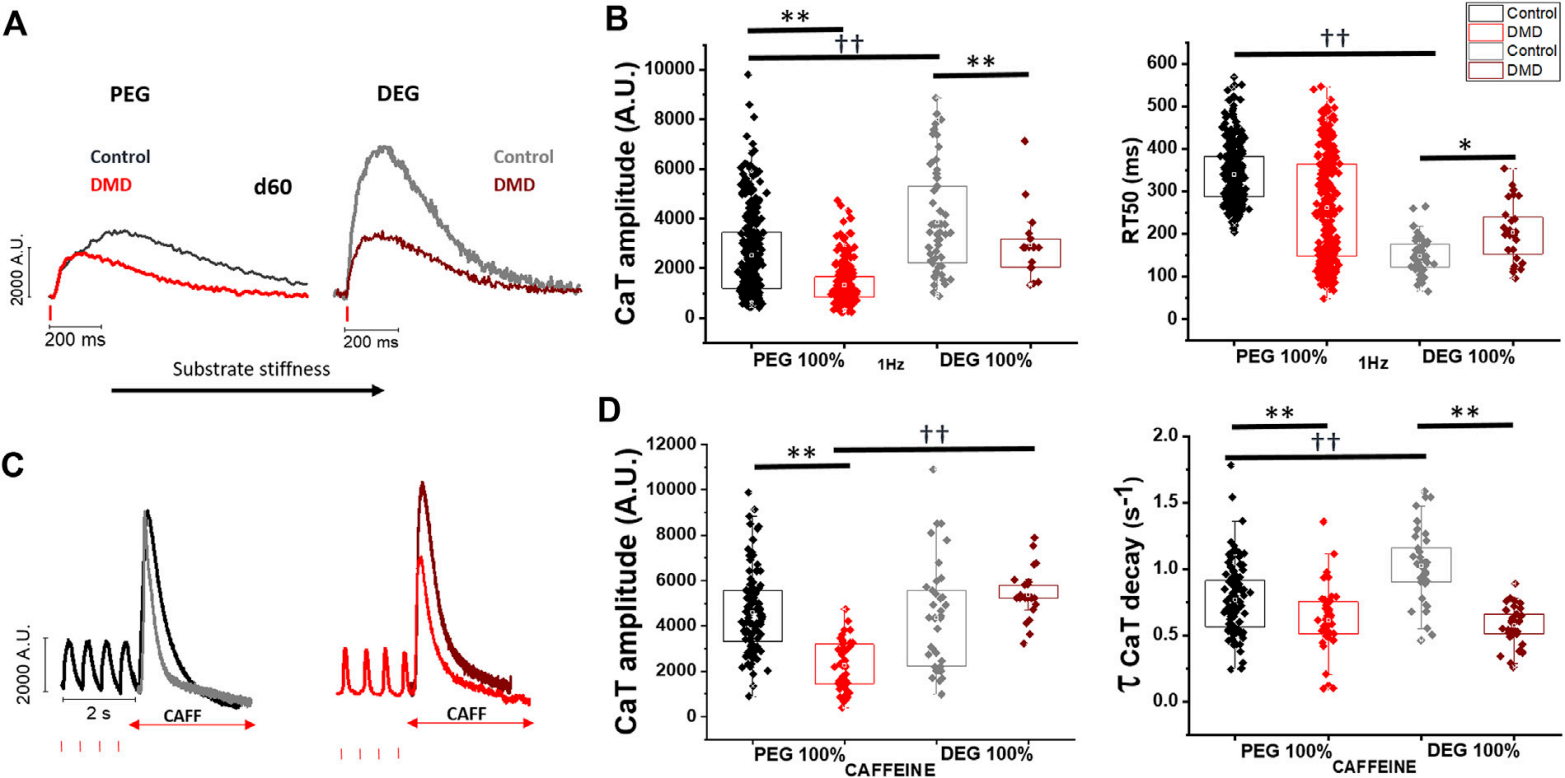
Impact of substrate stiffness on calcium transient amplitude and duration. To evaluate the impact of substrate stiffness on loss of full-length dystrophin, DMD- and control-CMs were compared for CaT amplitude and caffeine-evoked CaT on softer (PEG)- versus stiffer (DEG)-microgrooved surfaces (MPs) at 37°C at and external [Ca^2+^] = 1.8 mM.**(A)** Representative CaT profiles at day 60 and of CaT amplitude (Fluorescence Arbitrary Units, A.U.) and RT50 of CaT decay (ms) of DMD-versus control-CMs under softer or stiffer MPs. **(B)** Data are reported in box plots report control-versus DMD-CaT amplitude and RT50 (ms) on both softer or stiffer substrates (PEG: Control N = 3, *n* = 336; DMD N = 5, *n* = 251; DEG: Control N = 2, *n* = 50; DMD N = 3, *n* = 67). **(C)** Caffeine-induced CaTs (quick exposure to 10 mM Caffeine) after a series of 2 Hz paced CaTs. **(D)** Average of caffeine-induced CaT amplitude (Fluorescence Arbitrary Units, A.U.) was measured by localized caffeine exposure after steady-state calcium transients at 2 Hz prior. Caffeine transient CaT amplitude (CaTA CAFF/CaTA 2 Hz ratio) and decay (τ, s−1) of DMD- and control-hiPSC-CMs were compared on both PEG (softer) and DEG (stiffer) substrates. (PEG: Control N = 2, *n* = 83; DMD N = 2, *n* = 46; DEG: Control N = 2, n = 23; DMD N = 2, *n* = 21). Data were represented as a box (median [interquartile range]) and whisker plots. Supporting information given in Supplementary Table S1. One-way analysis of variance (ANOVA) with a Tukey post-hoc test with statistical significance set at *p < 0.05 and **p < 0.01 versus control and ††*p* < 0.01 versus internal substrate condition.

We tested a palette of micropatterned substrates with increasing stiffness to assess the impact of substrate stiffness on calcium handling in hiPSC-CM (Figure 5A). Mixing PEG with adjustments of the crosslinker diethylene glycol-diacrylate (DEG-DA) concentration (PEG:DEG %: 75:25, 50:50, 25:75 and DEG 100%) resulted in increased substrate stiffness (Figures 5B,C). Control hiPSC-CMs (day 60 post-differentiation) showed a gradual increase of Ca-T amplitude with the increase of the substrate stiffness (Figures 5D,E, Figures 6A,B), obtained by increasing the percentage of DEG-DA in the monomere mixture. The maximal amplitude was obtained using polyurethane (PUA)-based rigid substrates (Figure 5E). Moreover, the time to peak (TTP, ms) and the time to the 50% of CaT decay (RT50, ms) were faster in control cells grown on stiffer substrates (DEG 100%) as compared with those grown on softer patterns (PEG 100%, see Figures 6C,D). We then tested the effects of substrate stiffness on cell contractility (Supplementary Figure S3). In line with the effects on Ca-T amplitude, control hiPSC-CMs displayed higher % cell shortening on stiffer PEG substrates, compared to cells grown on DEG patterns (Supplementary Figure S3A). In further analysis, a pacing train protocol of 2 Hz followed by a rest pause of 10 s was applied to evaluate the post rest potentiation of cell shortening (Supplementary Figure S3B). The amplitude of post-rest potentiated beats was higher in cells grown on stiffer substrates, but the relative increase was similar when compared with cells grown on soft surfaces, suggesting a similar inotropic reserve (Supplementary Figure S3B).

### Stiffer substrates do not enhance calcium release in DMD hiPSC-CMs, despite a higher SR Ca content

Figure 6 reports the impact of stiffer substrates on age-matched control and DMD hiPSC-CMs. DMD-(hiPSC)-CMs grown on 100% DEG patterns displayed increased Ca-T amplitude when compared with the softer substrate (PEG 100%), (Figures 4A,B; Supplementary Figure S4). However, compared to controls, DMD-hiPSC-CMs had reduced Ca-T amplitude on both soft and stiff substrates. Moreover, in control cells the kinetics of Ca-T decay accelerated significantly in cells grown on 100% DEG substrates as compared with those grown on softer substrates (Figures 6A,B; Supplementary Figure S4). Contrarily, in DMD-CMs the kinetics of Ca-Ts did not change substantially on stiffer vs softer substrates (Figures 6A,B; Supplementary Figure S4).

To gain insights into the role of SR calcium content, we analyzed caffeine-evoked Ca-Ts (Figures 6C,D). In control-CMs, caffeine Ca-T amplitude was similar between the softer and stiffer substrate (Figure 6D). Additionally, caffeine Ca-T decay kinetics (tau, s^−1^) became faster in control-CMs grown on the stiffer substrate, suggesting increased capability of intracellular calcium removal by the sodium calcium exchanger (NCX). Conversely, in DMD-CMs caffeine-induced calcium transient amplitude on DEG surfaces was markedly increased when compared to the softer PEG substrates. Indeed, the amplitude of caffeine-induced Ca-Ts from DMD-CMs grown on stiffer substrates became similar to controls (Figure 6D). The decay kinetics (tau, s^−1^) of Caffeine-induced Ca-Ts was slower compared to controls, regardless of the substrate stiffness. Taken together, these results may indicate a reduced capability of intracellular calcium extrusion in DMD-CMs (Figure 4D) and increased diastolic Ca^2+^ levels (Supplementary Figure S5).

## Discussion

In striated muscle the full-length dystrophin (Dp427) network covers almost the entire cytoplasmic surface of the plasma membrane. Within the cytoskeletal lattice complex termed costameres, dystrophin serves as a shock absorber, promoting membrane stability and transduction of mechanical force from the extracellular matrix during muscle contraction or stretch (Rahimov and Kunkel, 2013). Calcium handling abnormalities have been described as a major consequence of dystrophin and other dilated cardiomyopathy (DCM)-related mutations, but the mechanisms linking dystrophin deficiency to abnormal calcium cycling remain unclear (Pioner et al., 2020).

For Duchenne Muscular Dystrophy (DMD), the two main hypotheses related to calcium handling abnormalities are membrane fragility/damage (Carson et al., 2016), as reported in the human DMD-hiPSC-cardiomyocyte (Guan et al., 2014; Macadangdang et al., 2015) and *mdx* mouse (Fanchaouy et al., 2009) model, or altered ion channel function with dysregulation of calcium homeostasis as a direct consequence of the altered dystrophin glycogen complex (DGC) (Zhan et al., 2014). In this work, we have studied the Ca-Ts and APs recorded from DMD hiPSC-CMs obtained from a patient with DMD-related cardiomyopathy, carrying a deletion of exon 50 (Δ exon 50) in *DMD* gene (Pioner et al., 2020). We compared these with the results obtained in control cell lines and a positive isogenic control created by CRISPR-Cas9 targeting the DMD gene (c.263delG), as previously described (Querceto et al., 2022). Previous reports using these DMD hiPSC-CM lines exhibited membrane fragility (Guan et al., 2014) and diminished structural/functional response to an underlying substrate with nanotopographic grooves compared to normal cardiomyocytes that was associated to a lower level of actin cytoskeleton turnover (Macadangdang et al., 2015). Moreover, we recently reported that absence of full-length dystrophin is sufficient to slow Ca-T kinetics, causing an overall impairment of contractility in single hiPSC-CM (Pioner et al., 2019b; Bremner et al., 2022). This work showed that highly matured DMD-hiPSC-CMs are capable to display mechanisms of contractile dysfunction that are independent to systemic alterations occurring *in vivo*. A major impact was attributed to cell growth on nanopatterned substrates compared to the commonly used flat glass coverslips. However, these findings suggested either retarded or altered maturation of cardiomyocyte structures associated with these functions. In this work, Ca-Ts were recorded from hiPSC-cardiomyocytes at specific time points of maturation (60, 75, 90 days from cardiac induction) on custom-made soft PEG-based micropatterned surfaces. We performed simultaneous recordings of APs and Ca-Ts with fluorescent indicators at the single cell level, to assess the key regulatory mechanisms of cardiac contraction of mature DMD-hiPSC-CMs (Pioner et al., 2019a). At any time point of maturation, DMD-hiPSC-CMs on PEG-substrates showed lower Ca-T amplitude as compared with controls. This is consistent with the reduced post rest potentiation of Ca-Ts, which suggested a reduced amount of calcium stored in the sarcoplasmic reticulum (SR) during the resting pause. This observation is also confirmed by the caffeine-evoked Ca-Ts, which demonstrated a strong reduction of SR-calcium content in DMD-hiPSC-CMs with respect to controls. In agreement, increased CaMKII activity and phosphorylation of RyR2 S2814 may be related to higher RyR open probability and to SR calcium leakage (Meyer et al., 2021) in DMD-hiPSC-CMs. Calcium leakage from the SR during diastole can partially account for the reduced Ca-T amplitude of DMD-hiPSC-CMs, as previously observed in the mdx mouse model (Ullrich et al., 2009; Ather et al., 2013; Kyrychenko et al., 2013). Previous studies have shown that the SR Ca^2+^ release mechanism through RyR is impaired in both cardiac and skeletal muscles in DMD (Bellinger et al., 2009; Fauconnier et al., 2010). In cardiac muscle, other studies showed that genetic inhibition of RyR2 phosphorylation at S2808 or S2814 can reduce RyR2 oxidation, suggesting a potential interaction between these posttranslational pathways (Kyrychenko et al., 2013; Wang et al., 2015). In addition, other authors identified S-nitrosylation and calstabin 2 as a cause of RyR2 Ca^2+^ leakage leading to sudden cardiac arrhythmias in mdx mice (Fauconnier et al., 2010).

In addition, we tested the function of the β-adrenergic pathway in DMD-hiPSC-CMs. Previous observations of the expression profile of β-adrenergic receptors (AR) in hiPSC-CM displayed a time-dependent increase of β_2_ and particularly β_1_ ARs (Jung et al., 2016). To avoid discrepancy related to different AR expression or reduced level of cAMP due high activity of phosphodiesterases (PDEs) (Giannetti et al., 2021), we used forskolin (FSK, an activator of adenylyl cyclase) to test the activation of the downstream pathway. In the presence of forskolin, Ca-Ts doubled in amplitude in both control and DMD, suggesting preserved positive inotropic responses. This is in keeping with the similar acceleration of Ca-T kinetics in DMD and CTR cells under FSK. Preserved phosphorylated RyR-S2808 level confirmed no alterations of the PKA phosphorylation target.

With simultaneous recording of voltage-sensitive and calcium fluorescent dyes, we observed that APs had a similar profile in control- and DMD-hiPSC-CMs and showed a longer plateau phase at later stages of maturation with respect to earlier stages. These results are in line with previous observations (Jelinkova et al., 2020) but do not exclude the presence of minor electrophysiological abnormalities in DMD-hiPSC-CMs, as previously reported (Eisen et al., 2019). On the other hand, Ca-T decay, which reflects the mechanisms of calcium recovery after contraction, was overall significantly faster in DMD vs control and became even faster at longer times in culture. While control cells display a gradual increase of Ca-T amplitude with advanced maturation, DMD-hiPSC-CMs do not show any significant enhancement of Ca release at later stages of maturation, suggesting a specific impairment of systolic Ca release. Interestingly, our data support the idea that a lesser maturation or impairment of the SR apparatus, preventing any increase of SR Ca storage with maturation, is responsible for the lack of Ca-T increase with increasing time in culture. Finally, we observed that cardiomyocytes obtained from the CRISPR-Cas9 gene edited cell line (c.263delG) recapitulated the features of calcium handling observed in the patient-specific cell line (Δ exon 50), as we previously observed (Macadangdang et al., 2015; Pioner et al., 2019b). These findings support the idea that smaller Ca-Ts are a stable alteration affecting developing cardiomyocytes lacking full-length dystrophin and this is associated with the reduced force generation reported in other DMD cell lines (Chang et al., 2021). However, shorter calcium transient duration was not expected, according to our previous finding on single cells (Pioner et al., 2019b) and other findings obtained in 3D tissues (Kyrychenko et al., 2017; Moretti et al., 2020) reporting slower calcium transients. However, most of the previous results were obtained with stiff substrates or matrixes (glass or polyurethane-based coverslides), which may change the kinetics of Ca-Ts, as we have clearly observed.

Indeed, we tested a palette of substrate materials with increasing stiffness obtained by changing the percentage of polyethylene glycol, (PEG-DA) and diethylene glycol, (DEG-DA). Increasing the amount of DEG led to stiffer substrates, the maximal stiffness was obtained with 100% DEG-DA. In the control cell lines, we observed a clear increase of basal and post rest Ca-T amplitude with the increase of substrate stiffness. In DMD-hiPSC-CMs, however, we observed a much smaller increase of Ca-Ts when cells were grown on DEG substrates. Moreover, on stiffer substrates, Ca-T decay became slower in DMD-hiPSC-CMs compared to control cells grown in the same conditions. In addition, slower caffeine-induced CaT decay suggested that DMD-hiPSC-CMs have reduced ability to extrude calcium suggesting reduced NCX function in DMD-CMs. Together with increased RyR open probability (due to CaMKII phosphorylation), this may increase diastolic Ca^2+^ levels and expose cardiomyocytes to a higher risk of cellular arrhythmias. All together, these results emphasize the idea that calcium transient dysregulation is a major disease mechanism in developing DMD cardiomyocytes. In addition, the reduced ability to extrude intracellular calcium is a possible additional defect reducing the adaptation of DMD-hiPSC-CMs to changes of the substrate stiffness. The mechanical stiffness of the surrounding extracellular matrix (ECM) critically determines normal cell function, stem cell differentiation and tissue homeostasis (Engler et al., 2006). Among these, cardiomyocytes actively probe the rigidity of their extracellular environment by exerting traction forces *via* transmembrane proteins named integrins (Santoro et al., 2019). However, it is still poorly understood how cardiac cells sense matrix stiffness and how they transduce the mechanical information into specific cellular responses or functional phenotypes (Querceto et al., 2022). Key proteins in this scenario may include dystrophin and the glycoproteic complex (DGC). In dystrophin-associated cardiomyopathies, i.e. Duchenne (DMD) and Becker (BMD) Muscular Dystrophies, caused by the absence or the decreased expression of full-length dystrophin, respectively, the sarcolemma becomes fragile and susceptible to damage. For instance, the lack of dystrophin leads to reduced expression of its binding partner dystroglycan, a key link to laminin. Glycosylation of alpha-dystroglycan is necessary for its binding to laminin (Fallon and McNally, 2018). For instance, when the external load varies due to an increase of the extracellular matrix stiffness from pathological remodeling (e.g., myocardial fibrosis), DMD cells would be less capable of adapting force production to the new environmental features, amplifying their contractile impairment.

### Study limitations

In this work, we explored another important feature of hiPSC-CMs, related to the ability of these cells to adapt their function in response to an external cue. These findings highlights that the maturation of cardiomyocytes is strongly influenced by the extracellular microenviroment. Using a time point analysis with different substrate conditions, we identified an important feature of dystrophic cardiomyocytes: e.g., reduced ability to adapt the regulation of calcium handling upon variation of the extracellular matrix load. The underlying substrates can mimic some aspects of cardiac extracellular matrix (cECM), e.g. anisotropy and stiffness. Substrates with low (e.g., PDMS, Matrigel, hydrogels) and high (e.g., glass, polyurethane) stiffness can provide different levels of external load, influencing the force of contraction and protein expression (Querceto et al., 2022). The current work used different substrates that are far from recapitulating the physiological range of extracellular tissue stiffness. For this reason, culture conditions mimicking the physiological stiffness of the cECM are needed to verify the role of cell/cECM. For instance, substrates with 10 vs 35 kPa purposed the idea that tissue stiffness may trigger mechanisms related to contractile dysfunction, oxidative stress and telomere shortening in DMD-hiPSC-CMs (including the c.263delG DMD line) (Chang et al., 2021). More physiological conditions should be applied to verify the impact of cardiac cell/extracellular matrix interaction (Bremner et al., 2022; Querceto et al., 2022). However, for 2D cell cultures, stiffer substrates are important to stress additional pathological mechanisms (Macadangdang et al., 2018). This is important to discern maturational alterations from maladaptive mechanisms. In addition, this work has evaluated only one of the CaMKII pathway targets (RyR phosphorylation site S2814) but future studies must consider a broader range of E-C coupling proteins. This can be feasible with novel Multi-omics analysis (e.g., transcriptomic, proteomic and/or metabolomic analysis). This work was limited to a previously characterized cell line to study the calcium transient alterations in DMD-null cardiomyocytes (Guan et al., 2014; Macadangdang et al., 2015; Macadangdang et al., 2018; Pioner et al., 2019b) and comparison was made with an unrelated healthy control cell line (Guan et al., 2014; Macadangdang et al., 2015; Pioner et al., 2019b) and its isogenic CRISPR-Cas9 edited cell line (c.263delG) (Pioner et al., 2019b). Isogenic CRISPR-Cas9 edited cell lines, either induced (DMD-null) in controls or corrected in patient cell lines (Zhang et al., 2022), are the most rigorous model to verify the phenotype caused by gene mutations. Future studies aimed to verify the impact of novel therapies should include multiple isogenic pairs to exclude bias due to interpersonal variability.

### Conclusion

Better understanding of the impact of maturation strategies, such as long-term cultures and variation of substrate stiffness on hiPSC-CM function will provide new insights to create microenvironment that may more closely recapitulate the ECM alterations occurring in several pathologies, including DMD. The mechanical properties of ECM induce an adaptive response within the cytoplasm and the nucleus, leading to changes in cell fate specification and function (Nakayama et al., 2014). Downregulation or total absence of proteins involved in the costamere domains cause dramatic defects in the mechanosensing and mechanotransduction in muscular dystrophies and cardiomyopathies (Ervasti, 2003; Querceto et al., 2022). In this context, hiPSC-CMs obtained from patients are the most suitable platform for the investigation of cell/ECM interaction. To this aim, the possibility of targeting genes of interest by CRISPR-Cas9 gene editing technology allows the validation of the pathogenicity of any cardiomyopathy-associated variant (Bhagwan et al., 2020; Pioner et al., 2020). Moreover, it may contribute to elucidate the role of specific proteins with debated function and involved in physiological responses during cECM stiffening. In this context, full-length dystrophin plays a crucial role to transmit mechanical signaling throughout extracellular and intracellular compartments. For this reason, the loss of full-length dystrophin and DGC (Duelen et al., 2022) in DMD-hiPSC-CMs can serve as a model to investigate the signaling between the extra- and intracellular cardiac space. The consequences of full-length dystrophin deficiency appear in the very early phases of cardiac cell/tissue development, likely even before cardiac formation (Jelinkova et al., 2019).

Ventricular tachyarrhythmias in patients with DMD can be caused by a combination of mechanisms, including conduction abnormalities, fibrosis and fatty replacement of the myocardium (Frankel and Rosser, 1976; Fayssoil et al., 2017; Pioner et al., 2020). In addition, enhanced triggered activity due to abnormal myocyte Ca^2+^ homeostasis can promote arrhythmias in patients with DMD. Although DMD patients typically develop cardiac symptoms including arrhythmias in the presence of a structured cardiomyopathy, our strategy used DMD-hiPSC-CMs gain insight into the interplay between causal and adaptive alterations of cardiac cells during the early-stages of disease progression over a longer period of time. This approach suggested that causal changes can presumably be identified from the beginning of cardiac differentiation, even before the pathology becomes manifest, while adaptive and maladaptive processes can influence the disease progression. For this reason, the natural myocardium stiffness and the increase of fibrosis at later stages of the disease can be major causes of dilated cardiomyopathy and sudden death in DMD patients.

## Methods

### Cardiac differentiation and single cell maturation

The isolation of the human cells and the subsequent reprogramming into iPSC lines was performed in conformation with the Declaration of Helsinki. Urine-derived cells from a healthy male donor into hiPSC lines (UC3-4 A1) using a lentiviral vector carrying Oct3/4, Sox2, Klf4 and c-Myc as previously described (Jelinkova et al., 2020). We used previously described cell lines from a DMD patient carrying a deletion of exon 50 in *DMD* gene (UC72039) and compared to a healthy control (UC3-4) (Guan et al., 2014) and its isogenic cell line (c.263delG, UC1015-6) previously generated from control cell line by CRISPR-Cas9-mediated deletion of a single frame at the 5′ of Exon1 in *DMD* gene (Pioner et al., 2019b). Both UC72039 and UC1015-6 were previously characterized for the absence of the full-length dystrophin (Dp427) (Guan et al., 2014; Macadangdang et al., 2015; Pioner et al., 2019b; Bremner et al., 2022). For cardiac differentiation we applied a monolayer directed differentiation protocol onto a Matrigel matrix (Matrigel^®^ hESC-Qualified Matrix, Corning^®^, New York city, NY, United States ), using the cardiac PSC Cardiomyocyte Differentiation Kit (Life Technologies, Thermo Fisher scientific, Carlsbad, CA, United States Thermo Fisher) following the manufacturer’s instructions as previously described (Pioner et al., 2019b). Briefly, hiPSCs were maintained under feeder-free conditions in mTeSR medium (Stem Cell Technolgies, Vancouver, Canada) on a Corning^®^ Matrigel matrix and regularly passaged every 4–5 days. For cardiac differentiation, hiPSC colonies with 70–80% confluency were chemically dissociated using 1× Tryple (Life Technologies, Thermo Fisher scientific, Carlsbad, CA, United States Life Technologies) city, state abbrev, country), suspended into mTeSR with 5 μM of ROCK inhibitor (Y27632) and seeded as single cells onto Matrigel-coated wells of a 24 wells plate at a cell density of 40,000 cell/well (24-well plate). At 70% of confluency (two to three days) the medium is changed to Cardiomyocyte Differentiation Medium A (referred as day 0) to start cardiac induction. Medium A is replaced after 2 days with Medium B and following other 2 days with Medium C for final differentiation. hiPSC are fed every other day with Medium C until spontaneously beating monolayers appear (day 8–10). At day 12 and for further hiPSC-cardiomyocyte (hiPSC-CM) maturation, Medium C is replaced with RPMI plus B27 supplement (Life Technologies, Thermo Fisher scientific, Carlsbad, CA, United States ).

### Maturation on PEG-DA or DEG-DA substrates micropatterned topography

On day 20 post differentiation, single hiPSC-CMs were obtained from beating monolayers. Beating monolayers were then pre-treated with 5 μM Y27632 in the RPMI/B27 medium for 1 h. For single cell dissociation, after careful medium wash with PBS, monolayers were incubated for enzymatic dissociation with Tryple (Life Technologies, Thermo Fisher scientific, Carlsbad, CA, United States Life Technologies) (10 min at 37°C). Plating media was composed by RPMI/B27 and 10 μM ROCK inhibitor. Single hiPSC-CMs were spun and sparsely seeded at the density of 20,000 cells/cm^2^ (at least 40,000 cell/substrate) onto fibronectin (Human Fibronectin Native Protein, Life Technologies, 10 μg/ml) substrates (25 mm of diameter) (PEG or DEG or commercial Polyurethane Acrylate (PUA)-based nanopatterned substrates purchased from CuriBio, Seattle, WA, United States ) in 6-well plate. Single hiPSC-CMs were fed every other day until experimental days. Dual recording experiments of long-term cultured hiPSC-CMs were performed at day 60, 75 and 90 p. d.

### Fabrication of PEG- and DEG-DA substrate with micropatterned topography

Micropatterned substrates were prepared by soft lithographic technique. A master sample was replicated by a PDMS (polydimethylsiloxane) mold that is used as template for the pattern replication as previously reported (Pioner et al., 2019a). All materials for micropatterning were purchased by Sigma Aldrich. Master fabrication, glass slide treatment and fabrication of PDMS, prior to patterned sample fabrication, followed a procedure that was described in a previous work (Pioner et al., 2019a). *Glass slides treatment*—Glasses to support the PEG patterns were silanized to prevent the peeling-off of the PEG layer during the cell culture. First, glasses were washed with an alkaline piranha solution (water, aqueous ammonia and hydrogen peroxide 5:1:1 v/v) at 70°C for 15 min. Then, the glass were rinsed with water and the isopropyl alcohol and, after drying, they were immersed in a solution of 3-(trimethoxysilyl) propyl methacrylate) (MAPTMS, 0.064 mM in ethanol) for 1 h. At the end, glasses were washed with isopropanol and dried.

*Fabrication of PDMS mold*—Monomeric PDMS mixture was prepared by mixing the two components of a commercially available PDMS kit (Sylgard 184) company, city, state abbrev, country) in a 10:1 w/w ratio (base and curing agent) and then casted on the silicon master. After curing at 100°C for 30 min, the crosslinked PDMS mold was peeled off by the master, as previously reported (Pioner et al., 2019a).

*PEG-DA and DEG-DA pattern printing*—A small amount (∼20 µL) of a solution of PEG-DA (M_n_ 250) (250 Mn) or DEG-DA and Irgacure 389 photoinitiator (1% w/w) was dropped on a silanized glass slides, Then, the PDMS mold was directly placed onto the surface. Irradiation by UV light at λ = 385 nm (M385CP1-C4, ThorLabs, Newton, NJ, United States ) for 10 min allowed the formation of the crosslinked PEG-DA or DEG-DA network. PDMS mold was gently peeled off from the substrates and used again after washing in water and methanol. Images of mold and micropatterned DEG-DA substrates were obtained by scanning electron microscopy (S.E.M.) (Figure 5B).

### Mechanical characterization of the polymer networks

Samples for dynamical mechanical analysis (DMA) were prepared by mixing PEGDA and DEGDA with 1% wt. Irgacure 389. The mixture was infiltrated by capillary force between two glass slides separated by a 200-um spacer. The monomer mixture was cured under UV light at 385 nm for 10 min at room temperature (following the curing procedure used for micropatterned sample fabrication). Strips (approximately 5 mm in length, 4.5–5 mm in width and ≈200 um in thickness) were clamped in tension mode in a DMA800 analyzer (Perkin Elmer) and analyzed in strain-control mode, imposing a 0.020 mm strain with 1 Hz frequency, in the -20C-120°C temperature range. Temperature ramp was set at 2.5°C/min. The mechanical characterization was repeated three times for PEG 100% and DEG 100% samples, which are respectively composed of PEG-DA and DEG-DA. The value of the storage modulus at E′ was obtained by calculating the integral mean of the E′-T curve between 36°C and 38°C and averaging the results. Values are expressed as mean ± SEM.

### Dual recording of action potential and calcium transient

For dual recordings of APs and CaTs, hiPSC-CMs were loaded with 2 μL/ml Fluovolt (Thermo Fisher, Waltham, MA, United States Thermo Fisher), 2 μL/ml of Cal630 (AAT Bioquest, Sunnyvale, CA, United States ) and 5 μL of Power Load™ concentrate (Thermo Fisher, Waltham, MA, United States ) for 30 min at 37 °C and then washed with pre-warmed culture media before placing the cover slide into the experimental chamber. The experimental chamber features platinum electrodes for electrical field stimulation, connected to a stimulator (DigiTimer, Welwyn Garden City, United Kingdom) delivering short (3 ms) voltage pulses. During measurements, cells were continuously perfused with heated Tyrode buffer (in mM: NaCl 138, KCl 3.7, HEPES 20, KH_2_PO_4_ 1.2, MgSO_4_·7H_2_O 1.2, Glucose 5, CaCl_2_·2H_2_O 1.8, pH 7.0) to keep the temperature stable at 37 ± 1°C. For fluorescence studies, cells were simultaneously illuminated by LED light at two different wavelengths, blue (488 nm) for excitation of Fluovolt and yellow (580 nm) for Cal630 dye excitation, using a multi-led system (Lumencor SPECTRA X, Beaverton, OR, United States ). A dual-wavelength band-pass filter cube (Semrock, IDEX, Lake Forest, IL, United States ) city, state abbrev, country) was used to allow fluorescence light from the two dyes to be collected by a single camera (Photometrics Prime sCMOS, Teledyne, Tucson, AZ, United States ): in particular, the filter allowed green light (515–545 nm, emission of Fluovolt) and red light (615–655 nm, emission of Cal630) to be collected. In order to separate the two emission wavelengths, we used an OptoSplit II light splitter (Cairn Research Ltd., Kent, UKCairn) that was able to separate the two spectral components of the fluorescence image and focus them simultaneously on the upper and lower half of the camera chip. MetaMorph software (Molecular Devices, San Jose, CA, United States ) was used to collect and analyze fluorescence images. The camera collected images at an average rate of 90 frames per second. In each selected microscope view field, a number of single hiPSC-CMs were selected and chosen as regions of interest. The background-corrected average fluorescence values from the pixels in each selected region of interest (myocyte) were recorded at each of the two wavelengths under different stimulation conditions for 5–10 s in each condition. For the analysis of action potential or calcium transient kinetics during steady-state stimulation, the average of 5–10 subsequent AP or CaT traces was calculated to reduce noise. For Figures 1B,D, the exponential curves with Stirling’s approximation were obtained by fitting the mean values of CaT amplitude at day 60, 75 and 90 days post differentiation using the following equation: y = a + b*(exp (k*x)^−1^)/k. For testing β-adrenergic response, control and DMD hiPSC-CMs were recorded on the same cells before and after incubation with forskolin (FSK, 1 μM) for at least 10 min. Calcium transients were analyzed as fluorescent intensity emission detected by the photomultiplier with subtraction of the background noise and expressed as fluorescent arbitrary units (A.U.). As reported in Supplementary Figure S6, there is no correlation (negative linear regression, Pearson’s correlation, *r* ^2^) between calcium transient amplitude (A.U.) and the selected cell area (pixels).

### Caffeine induced calcium transients

Caffeine-induced CaTs (quick exposure to 10 mM caffeine) were recorded in localized area with a double-barrel pipette with rapid change (<3 ms) following this sequence: series of 2 Hz paced CaTs—pacing stop and quick caffeine exposure, washout and series of 2 Hz paced CaTs. Caffeine transient CaT amplitude (A.U.) and decay (τ, s^−1^) of hiPSC-CMs were calculated.

### Cell fractional shortening

Single control hiPSC-CM (WTC11 and UC3-4) fractional shortening was visualized using a Nikon TS100 inverted microscope coupled to a video-based edge detection system at ×40 of magnification (Olympus). For measures of single hiPSC-CMs cultured for 60 days, at 20 days post differentiation, single hiPSC-CMs were re-plated onto 10 μg/ml human fibronectin-coated surfaces (35 mm diameter, Fisher). All cells were perfused at 37°C with a Tyrode solution subjected to a sequential pacing train of at least 10 s at 1 Hz. Traces were analyzed using the IonWizard (IonOptix) software. A minimum of five traces were analyzed and averaged for each cell. Spontaneously beating hiPSC-CMs were excluded from this analysis.

### CaMKII and RyR phosphorylation western blot

Western blot analysis was performed by a standard method on proteins isolated from monolayers from control- and DMD-hiPSC-cardiomyocytes, as previously described (Ferrantini et al., 2017). The following antibodies were used: p-RYR2 S2808 (Badrilla); p-RYR2 S2814 (Badrilla); p-CAMKII (Santa Cruz); CAMKIIδ (Abcam); GAPDH (Millipore). Relative intensity of individual bands from Western blots was quantitated using ImageJ software and normalized to GAPDH or total CaMKII (Supplementary Figure S7).

### Statistics

All data are reported as mean ± SEM and were compared using a one-way or two-way analysis of variance (ANOVA). Tukey post hoc test with statistical significance set at *p < 0.05 and **p < 0.01 were applied for differences in means between groups/conditions. The interquartile range test was performed for data distribution and selection. For each analysis, n represented number of cardiomyocytes and N the total number of cell differentiation runs from individual hiPSC passages or individual patients. The number of individual experiments (individual coverslides) for each assessment was at least two from different differentiation runs. Supporting information of results is reported in Supplementary Table S2.

## Data Availability

The original contributions presented in the study are included in the article/Supplementary Material, further inquiries can be directed to the corresponding authors.

## Abbreviations

AP, Action potential; APD, Action potential duration; Ca^2+^, Calcium; CAFF, caffeine; CaMKII, Ca^2+^/calmodulin-dependent protein kinase II; cAMP, Cyclic adenosine monophosphate; CaTA, Calcium transient amplitude; Ca-Ts, Calcium transients; cECM, Cardiac extracellular matrix; CM, Cardiomyocyte; DEG-DA, di(ethylene glycol) diacrylate; DMD, Duchenne Muscular Dystrophy; FSK, Forskolin; GAPDH, Glyceraldehyde-3-phosphate dehydrogenase; hiPSC, Human induced pluripotent stem cell; NCX, Sodium calcium exchanger; p.d, Post differentiation; PDMS, polydimethylsiloxane; PEG-DA, polyethylene glycol diacrylate; PKA, Protein kinase A; RT50, Time to 50% relaxation; RyR2, Ryanodine receptors two; S.E.M, Scanning Electron Microscopy; SERCA, Sarco-Endoplasmic Reticulum Calcium ATPase-2A; SR, Sarcoplasmic Reticulum; TTP, Time to peak; τ, Tau, calcium transient decay constant.
