# Corrigendum: Calcium handling maturation and adaptation to increased substrate stiffness in human iPSC-derived cardiomyocytes: the impact of full-length dystrophin deficiency

**DOI:** 10.3389/fphys.2023.1222400

**Published:** 2023-06-13

**Authors:** Josè Manuel Pioner, Lorenzo Santini, Chiara Palandri, Marianna Langione, Bruno Grandinetti, Silvia Querceto, Daniele Martella, Costanza Mazzantini, Beatrice Scellini, Lucrezia Giammarino, Flavia Lupi, Francesco Mazzarotto, Aoife Gowran, Davide Rovina, Rosaria Santoro, Giulio Pompilio, Chiara Tesi, Camilla Parmeggiani, Michael Regnier, Elisabetta Cerbai, David L. Mack, Corrado Poggesi, Cecilia Ferrantini, Raffaele Coppini

**Affiliations:** ^1^ Department of Biology, University of Florence, Florence, Italy; ^2^ Department of Neurofarba, University of Florence, Florence, Italy; ^3^ Department of Experimental and Clinical Medicine, University of Florence, Florence, Italy; ^4^ European Laboratory for Non-linear Spectroscopy (LENS), University of Florence, Florence, Italy; ^5^ Istituto Nazionale di Ricerca Metrologica (INRiM), Turin, Italy; ^6^ Department of Molecular and Translational Medicine, University of Brescia, Brescia, Italy; ^7^ National Heart and Lung Institute, Imperial College London, London, United Kingdom; ^8^ Unit of Vascular Biology and Regenerative Medicine, Centro Cardiologico Monzino IRCCS, Milan, Italy; ^9^ Department of Biomedical, Surgical and Dental Sciences, University of Milan, Milan, Italy; ^10^ Department of Chemistry “Ugo Schiff”, University of Florence, Florence, Italy; ^11^ Department of Bioengineering, University of Washington, Seattle, WA, United States

**Keywords:** human iPSC derived cardiomyocytes, dystrophin (DMD), substrate stiffness, calcium handing, duchenne muscular dystrophy (DMD)

In the published article, there was an error in the **Funding** statement. “This research received fundings from: Telethon Italy under grant agreement, grant number GGP16191 (CF), Telethon-UILDM, grant number GUP19012 (CF and GP) and by Ente Cassa di Risparmio di Firenze, grant number 2018.0987 (CF). This research received fundings from: Telethon Italy under grant agreement, grant number GGP16191 (CF), Telethon-UILDM, grant number GUP19012 (CF and GP) and by Ente Cassa di Risparmio di Firenze, grant number 2018.0987 (CF). We also thank MIUR Italy (“Progetto Dipartimenti di Eccellenza 2018–2022” allocated to Department of Chemistry “Ugo Schiff” and to the Department of Experimental and Clinical Medicine). We also thank MIUR Italy (“Progetto Dipartimenti di Eccellenza 2018–2022” allocated to Department of Chemistry “Ugo Schiff” and to the Department of Experimental and Clinical Medicine)”.

The correct **Funding** statement appears below.

