# Supplementary material for: Calcium handling maturation and adaptation to increased substrate stiffness in human iPSC-derived cardiomyocytes: The impact of full-length dystrophin deficiency
Source: Front Physiol. 2022 Nov 7;13:1030920. doi: 10.3389/fphys.2022.1030920 (PMC9676373; doi:10.3389/fphys.2022.1030920)
Supplement: Supplementary file 1 [file DataSheet1.pdf]

Calcium handling maturation and adaptation to increased substrate stiffness in human iPSC-derived cardiomyocytes: the impact of full-length dystrophin deficiency

Josè Manuel Pioner[1*#], Lorenzo Santini[2*], Chiara Palandri[2*], Marianna Langione[3], Bruno Grandinetti[4], Silvia Querceto[4], Daniele Martella[4,5], Costanza Mazzantini[2], Beatrice Scellini[3], Lucrezia Giammarino[2], Flavia Lupi[4], Francesco Mazzarotto[6,7], Aoife Gowran[8], Davide Rovina[8], Rosaria Santoro[8], Giulio Pompilio[8,9], Chiara Tesi[3], Camilla Parmeggiani[4,10], Michael Regnier[11], Elisabetta Cerbai[2], David L. Mack[11], Corrado Poggesi[3], Cecilia Ferrantini[3#] and Raffaele Coppini[2]

[1]Department of Biology, University of Florence, Florence, Italy [2]Department of Neurofarba, University of Florence, Florence, Italy [3]Department of Experimental and Clinical Medicine, University of Florence, Florence, Italy [4]European Laboratory for Non-Linear Spectroscopy (LENS), University of Florence, Sesto Fiorentino, Italy, [5]Istituto Nazionale di Ricerca metrologica (INRiM), Italy [6]Department of Molecular and Translational Medicine, University of Brescia, Brescia, Italy [7]National Heart and Lung Institute, Imperial College London, London, United Kingdom [8]Unit of Vascular Biology and Regenerative Medicine, Centro Cardiologico Monzino IRCCS, Italy, [9]Department of Biomedical, Surgical and Dental Sciences, University of Milan, Italy [10]Department of Chemistry "Ugo Schiff", University of Florence, Sesto Fiorentino, Italy [11]Department of Bioengineering, University of Washington, Seattle, WA, USA

*These authors equally contributed #Corresponding authors

SUPPLEMENTARY FIGURES

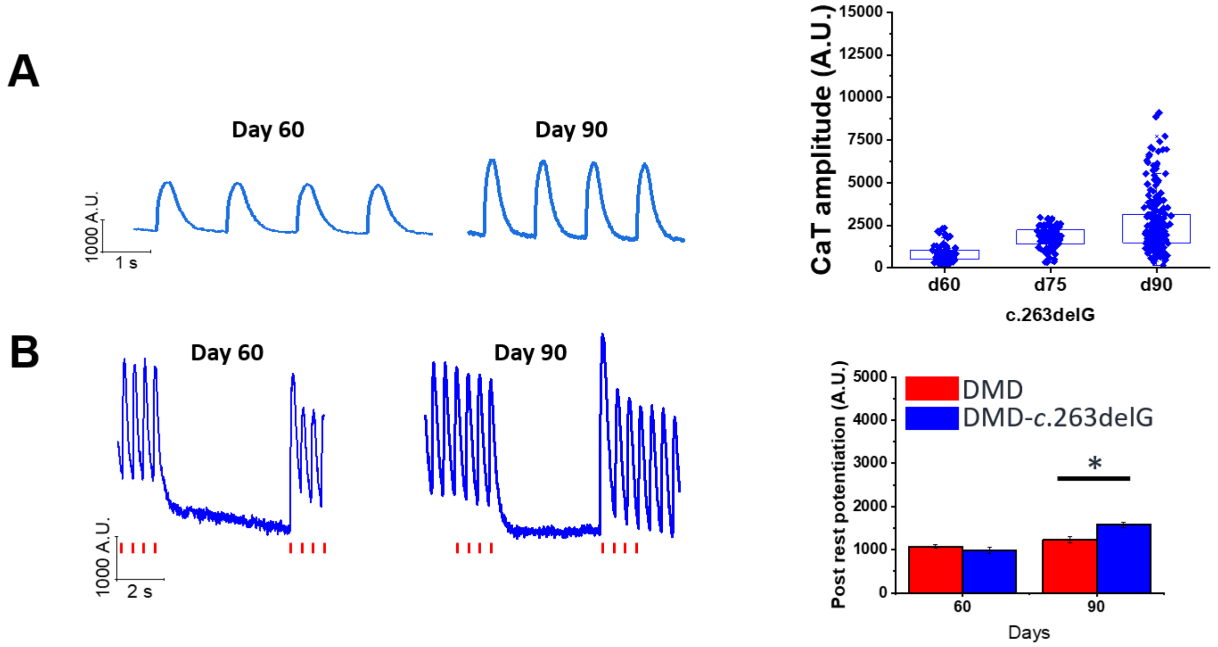

Supplementary Figure 1. Calcium transients in the CRISPR-Cas9 edited cell line (DMD-c.263delG). Calcium transients were estimated at day 60, 75 and 90 post differentiation at 37°C, 1.8mM [Ca^{2+}]. (A) Representative CaT profiles at day 60 and 90 and average CaT amplitude (Fluorescence Arbitrary Units, A.U.) of c.263delG versus DMD-CMs at day 60,75 and 90. (B) Sarcoplasmic reticulum (SR) contribution in calcium handling maturation was tested by a post rest potentiation protocol at multiple maturation time-points. The potentiation is expressed as the % of increase of the first post-rest CaT with respect of CaT pacing train before the pause (%). Post rest potentiation of c.263delG versus DMD-CMs is estimated at day 60 and day 90. c.263delG d60 N=2, n=81; d75 N=2, n=259; d90 N=2; n=260; DMD d60 N=3, n=193, d75 N=4, n=292; d90 N=4, n=169. One-way analysis of variance (ANOVA) with a Tukey post-hoc test with statistical significance set at * $p < 0.05$ and ** p 0.01 versus DMD-CMs.

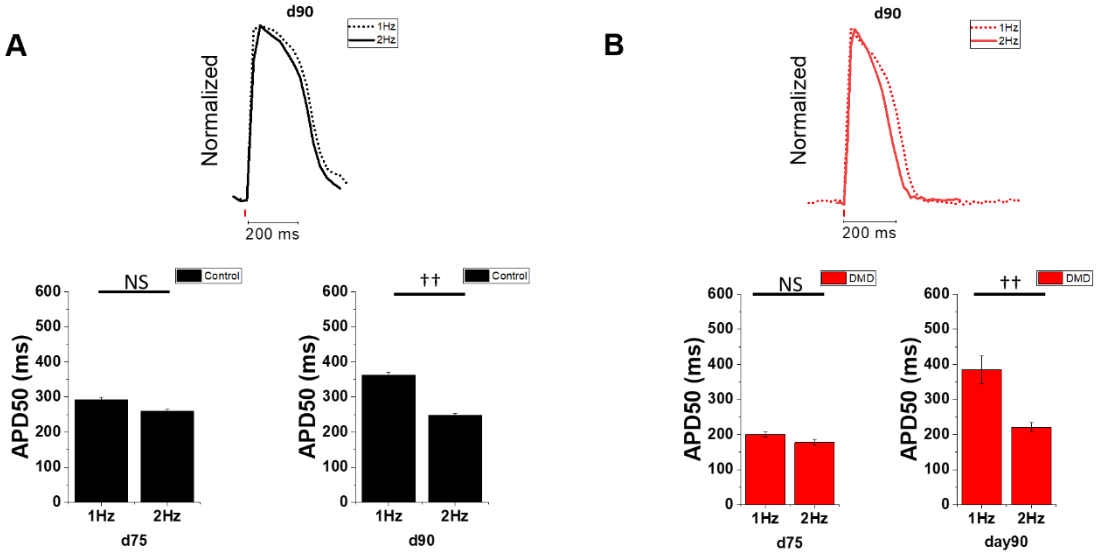

Supplementary Figure 2. Rate adaptation of action potential duration in late-stage hiPSC-CMs. Superimposed action potential (AP) profile of hiPSC-CMs was recorded both at 1 and 2 Hz to evaluate action potential duration (APD50, ms) and the response to frequency changes at both day 75 (Control N=2, n=186; DMD N=2; n=91) and 90 (Control N=2, n=119; DMD N=2; n=44). Data were represented as a box plots. † $p < 0.05$, †† $p < 0.01$ or NS for not significant versus 1Hz.

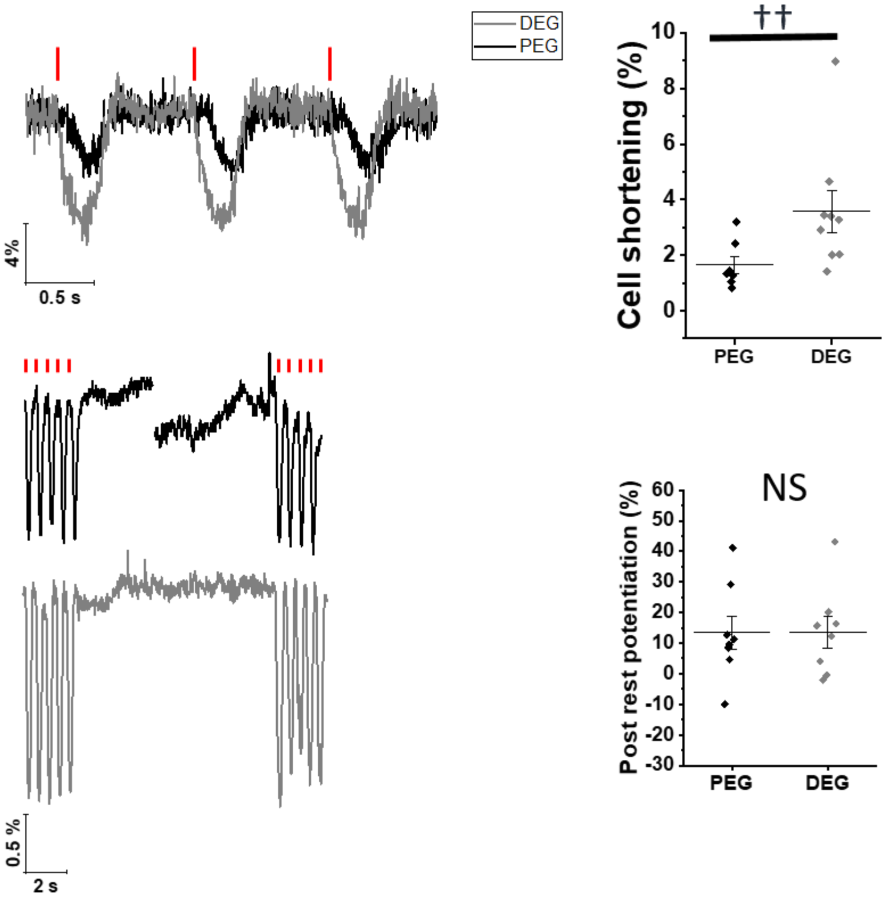

Supplementary Figure 3. Impact of substrate stiffness on cell contractility in control hiPSC-CM. (A) Cell fractional shortening of control-CMs on PEG and DEG-based micropatterned surfaces. Mean±SEM of cell contractility at 1Hz are expressed as percentage of shortening from relaxed cell length (%) (PEG: N=2; n=7); DEG: N=2; n=7). (B) Post rest potentiation of cell contractility was estimated from the percentage of increase of first twitch (%) after a resting pause of 10 seconds compared to a series of 2Hz paced CaTs (PEG: N=2; n=7; DEG: N=2; n=7. Data are reported as means ± SEM; one-way analysis of variance (ANOVA) with a Tukey post-hoc test with statistical significance set at † $p < 0.05$ and †† $p < 0.01$; NS not significant. Supporting information given in Table S1. N = number of differentiations; n = cells.

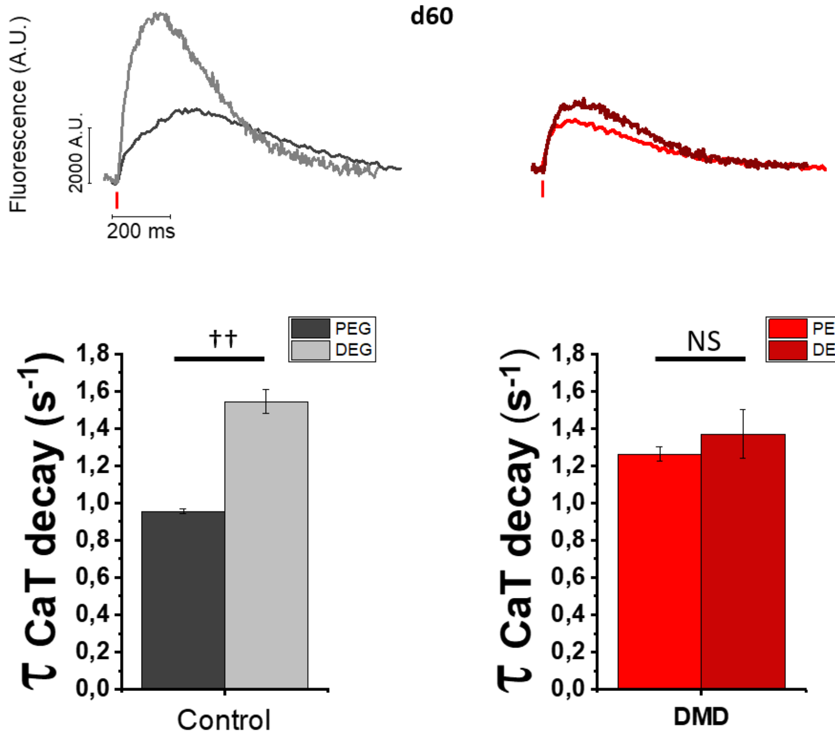

Supplementary Figure 4. Ca-transient decay in control and DMD-hiPSC-CMs grown on PEG vs. DEG substrates. The impact of substrate stiffness in DMD(hiPSC)-CMs was tested for CaT amplitude decay (τ, s^{-1}) on 100% polyethyleneglycole (PEG) vs 100% dyethylenglycole (DEG)-based microgrooved surfaces at 37 °C at and external $[Ca^{2+}]$ = 1.8 mM. (A) Representative CaT profiles at day 60 (Fluorescence Arbitrary Units, A.U.)) of control- and DMD-hiPSC-CMs. (B) Data were represented as a box plots. † $p < 0.05$, †† $p < 0.01$ PEG versus DEG condition.

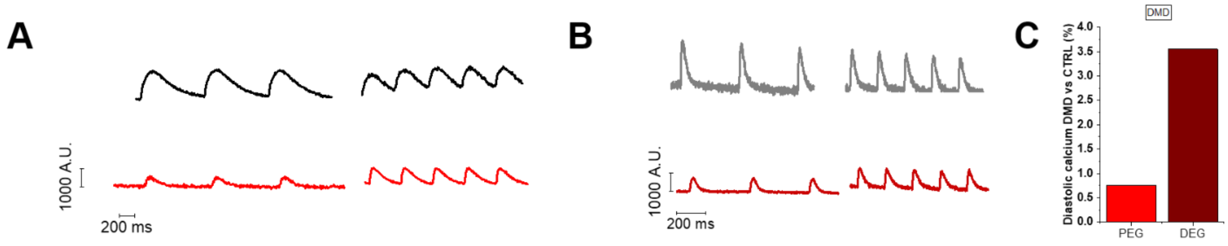

Supplementary Figure 5. Impact of substrate stiffness on resting intracellular [Ca^{2+}] in hiPSC-CMs. A) Representative simultaneous fluorescence recordings of intracellular [Ca^{2+}] from day 60 DMD- vs. Control-hiPSC-CMs at 1 vs 2 Hz of field stimulation on PEG B) and DEG substrates. C-D) Resting calcium level was estimated from the ratio between the baseline level at 1Hz vs 2Hz from DMD and controls on PEG and DEG substrates. One-way analysis of variance (ANOVA) with a Tukey post-hoc test with statistical significance set at * $p < 0.05$ and ** $p < 0.01$ versus control.

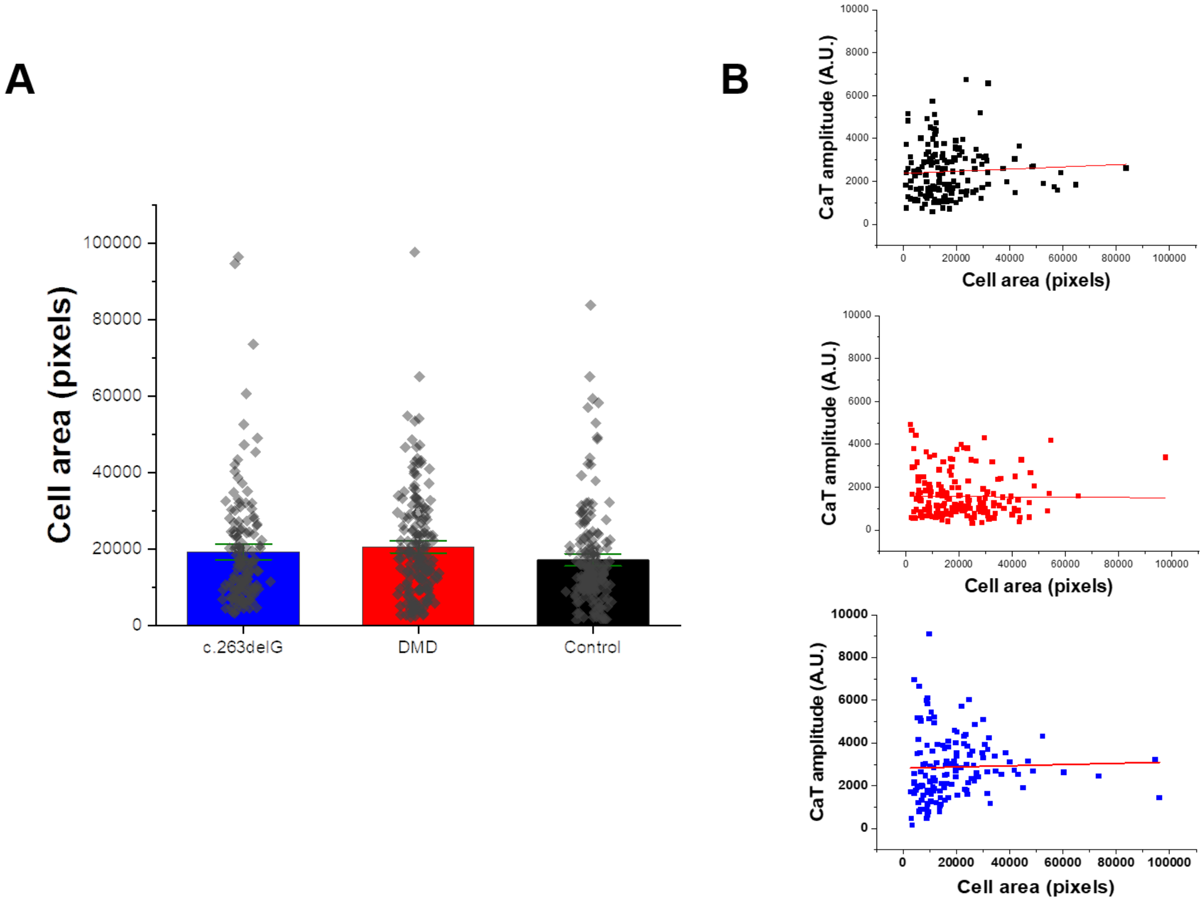

Supplementary Figure 6. Correlative analysis of Ca-transient amplitude and cell area. Correlative analysis of calcium transient transients (Fluorescence Arbitrary Units, A.U.) and hiPSC-CM area (pixels). A) Selected cell area (pixels) of c.263delG-, DMD- and control- cell lines during the dual recording of action potential and calcium transients (at day 90 post differentiation). Data were represented as a box plots with mean±SEM. B) Pearson correlation coefficient (r^2) was estimated by linear regression (red line) to correlate CaT amplitude (A.U.) against cell area (pixels) at day 90 Control- vs DMD vs c.263delG- hiPSC-CMs and ($p < 0.05$).

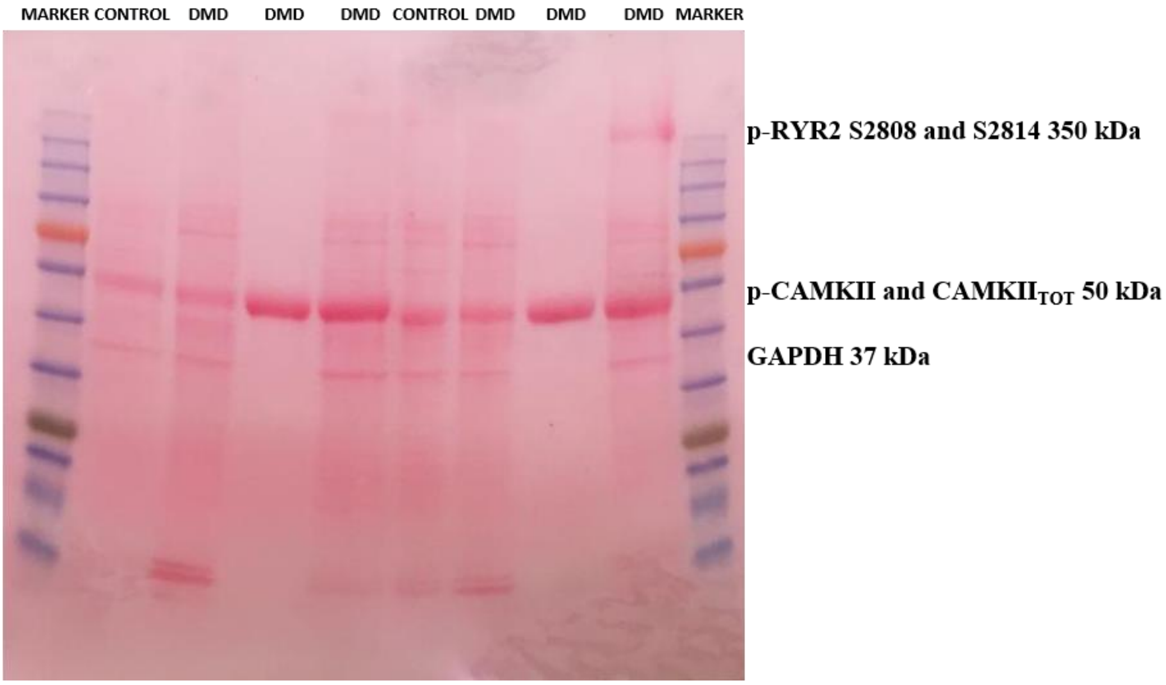

Supplementary Figure 7. Representative western blot staining for Ryanodine receptors (RyR2) phosphorylation sites (S2808/S2814, 350 kDa), total CaMKII and phosphorylated CaMKII (50 kDa), GAPDH (37 kDa) in DMD- vs. Control-hiPSC monolayers.

hiPSC-CMs	Cell line	day 60	day 75	day 90
**APD50 (ms)**	Control	168±65	291±7	362±9
	DMD	150±5	201±8	384±4
**CaT amplitude (A.U.)**	Control	2515±93	2036±73	4506±288
	DMD	1325±51	2245±76	1557±74
**CaT TTP (ms)**	Control	234±3	181±5	188±6
	DMD	150±6	134±4	129±4
**CaT RT50 (ms)**	Control	340±4	315±7	277±7
	DMD	245±8	283±5	212±6
**PR APD50 (ms)**	Control	418±4	400±1	380±2
	DMD	178±3	241±6	330±4
**PR RT50 (ms)**	Control	483±11	388±3	334±2
	DMD	366±17	417±3	435±8
**CaT PRP (A.U.)**	Control	2335±161	1917±91	4380±205
	DMD	1074±46	1745±78	1231±65

Supplementary Table 1. Summary of action potential and calcium transient results from control and DMD-hiPSC-CMs. Time point experiments of dual recording are reported for hiPSC-CMs at 1Hz of pacing rate and post rest (2Hz). Data are reported as Mean±SEM.

hiPSC-CMs	Cell line	PEG 100%	DEG 100%
**CaT amplitude (A.U.)**	Control	2515±93	3868±32
	DMD	1325±51	2992±49
**CaT TTP (ms)**	Control	234±3	130±7
	DMD	150±6	201±17
**CaT RT50 (ms)**	Control	340±4	149±6
	DMD	245±8	205±16

Supplementary Table 2. Summary of calcium transient exposed to micropatterned substrates with different stiffness. Results are reported for age-matched (day 60) control- and DMD-hiPSC-CMs at 1Hz of pacing rate on micropatterned (PEG 100% and DEG 100%) substrates. Data are reported as Mean±SEM.

c.263delG	day 60	day 75	day 90
**CaT amplitude (A.U.)**	839±67	1778 ±65	2541±101
**CaT TTP (ms)**	193±6	189±4	195±4
**CaT RT50 (ms)**	256±8	307±5	328±6

Supplementary Table 3. Summary of action potential and calcium transient results from c.263delG-hiPSC-CMs. Time point experiments of dual recording are reported for hiPSC-CMs at 1Hz of pacing rate. Data are reported as Mean±SEM.
